# Effects of visual art therapy on cognitive function in older adults: a systematic review and meta-analysis

**DOI:** 10.3389/fpsyt.2026.1828257

**Published:** 2026-08-19

**Authors:** Zhenhua Guo, Yingcong Liu, Shan Zhu, Yupeng He

**Affiliations:** 1 Department of Design, Dong-A University, Busan, Republic of Korea; 2 School of Music Education, Wuhan Conservatory of Music, Wuhan, Hubei, China; 3 School of Pharmaceutical Sciences, Wenzhou Medical University, Wenzhou, Zhejiang, China

**Keywords:** activities of daily living, cognitive function, language function, meta-analysis, older adults, systematic review, visual art therapy

## Abstract

**Objective:**

To systematically review the evidence on the effects of visual art therapy on global cognitive function in older adults, and to examine its impact on verbal fluency, naming ability, and activities of daily living.

**Methods:**

A systematic search was conducted in PubMed, Web of Science, Embase, the Cochrane Library, China National Knowledge Infrastructure (CNKI), the Chinese Biomedical Literature Database, Wanfang Database, and VIP Database for randomized controlled trials (RCTs) published from database inception to July 2026. Meta-analysis was performed using RevMan 5.4 and Stata 15.0. The primary outcome was global cognitive function, and secondary outcomes included the Verbal Fluency Test (VFT), the Boston Naming Test (BNT), and activities of daily living. Fixed-effects or random-effects models were used according to heterogeneity, and subgroup analyses were conducted.

**Results:**

A total of 10 randomized controlled trials involving 628 older participants were included, of whom 318 were allocated to visual art therapy and 310 to control conditions. Meta-analysis showed that visual art therapy significantly improved global cognitive function in older adults (SMD = 0.39, 95% CI: 0.23–0.54, P < 0.00001). For secondary outcomes, compared with control groups, statistically significant improvements were observed in verbal fluency (MD = 1.21, 95% CI: 0.12–2.29, P = 0.03), naming ability (MD = 1.74, 95% CI: 0.20–3.28, P = 0.03), and activities of daily living (SMD = −0.36, 95% CI: −0.62 to −0.10, P = 0.006) in the visual art therapy group. However, the evidence is based on only two or three studies and was rated as low or very low certainty. Subgroup analyses indicated larger effect sizes for interventions delivered 13–24 times, with 30–40 minutes per session, and among older adults with mild cognitive impairment, given the limited number of studies per subgroup, these findings are exploratory and should be interpreted with caution.

**Conclusion:**

Visual art therapy can significantly enhance global cognitive function in older adults and confers additional benefits on language functions and activities of daily living. As a safe, non-invasive, non-pharmacological intervention, visual art therapy may serve as an effective adjunctive approach for managing cognitive decline and daily functioning in the older population.

**Systematic review registration:**

https://www.crd.york.ac.uk/PROSPERO/view/CRD420261337553, identifier CRD420261337553.

## 1 Introduction

Global population aging is accelerating. In 2024, individuals aged 60 years and older accounted for approximately 10.3% of the world’s population. By 2050, this proportion is projected to reach nearly 22%, corresponding to 2.1 billion people (1). With shifts in population demographics, age-related cognitive decline is increasingly recognized as a major global public-health challenge (2). Cognitive decline is primarily manifests as deterioration in memory, language, attention, executive function, and judgment (3). When the degree of decline is sufficient to interfere with daily life, it is classified as cognitive impairment. Dementia, a severe form of cognitive impairment, currently affects approximately 56.9 million people worldwide and is projected to exceed 137 million by 2050 (4). Current clinical treatment strategies for cognitive decline rely primarily on pharmacotherapy, including cholinesterase inhibitors and memantine (5). Although these pharmacological agents have shown efficacy in mitigating cognitive decline, their effects exhibit substantial inter-individual variability and are frequently accompanied by side effects, adverse reactions, and risks of complications (6). This is particularly concerning in older adults, among whom multimorbidity and polypharmacy are highly prevalent and harmful drug-drug interactions cannot be overlooked (7). Consequently, the development of safe, scalable, and sustainable non-pharmacological interventions to support healthy cognitive aging and potentially modify cognitive decline across multiple functional domains has become an important research focus (8).

In recent years, visual art therapy has received increasing attention as a non-pharmacological intervention for older adults, owing to its potential to enhance cognitive function and quality of life. Visual art therapy is a process of spontaneous or guided creative expression that uses mixed media (e.g., painting, collage, sculpture) (9). Neuroimaging studies indicate that engaging in creative visual activities can modify activation patterns and functional connectivity in neural networks associated with attention, memory, and self-referential processing, including the default mode network (DMN) and the executive control network (ECN) (10–12). In addition, such activities may enhance oscillatory activity in the prefrontal and parietal cortices, thereby improving neural efficiency, abstract thinking, and executive control (13–15) and promoting the coordinated development of language, emotional regulation, and social interaction (16). Empirical studies in older adults further suggest that, by integrating memory, language, and planning abilities into meaningful, goal-directed creative tasks, visual art therapy not only significantly improves global cognitive function (17) but also concurrently enhances mood and social engagement (18), thereby helping older adults maintain functional independence in daily life (19).

Several systematic reviews have attempted to synthesize the evidence on visual art therapy; however, important limitations remain regarding study populations, intervention approaches, treatment parameters, and outcome measures. Specifically, the following issues have been identified: (1) insufficient methodological rigor in study selection, with some reviews pooling randomized controlled trials with uncontrolled or simple pre-post studies, thereby weakening internal validity and the robustness of causal inference; (2) language restrictions, as some reviews included only English-language studies and did not incorporate Chinese-language literature, potentially affecting the completeness of the evidence; (3) lack of clear specification of optimal intervention parameters (e.g., session duration and frequency), hindering precise evaluation of treatment effects; (4) substantial heterogeneity in intervention protocols, and the comparative effectiveness of visual art therapy alone versus visual art therapy combined with other art-based interventions remains unclear; (5) the potential influence of different living environments (e.g., nursing homes vs. community settings) on intervention outcomes requires further investigation; and (6) differences in treatment effects across population subgroups remain to be clarified.

Accordingly, this study conducted a systematic review and meta-analysis of randomized controlled trials (RCTs), in which global cognitive function was defined as the primary outcome, while language function and activities of daily living were treated as secondary outcomes. In addition, subgroup analyses were performed to further examine the potential influence of intervention modality, participant characteristics, and treatment parameters on intervention efficacy, with the aim of providing evidence-based support for the application of visual art therapy in older adults.

## 2 Methods

### 2.1 Protocol registration

This systematic review and meta-analysis was registered in the International Prospective Register of Systematic Reviews (PROSPERO) under registration number CRD420261337553.

### 2.2 Search strategy

A systematic literature search was conducted in the following electronic databases: China National Knowledge Infrastructure (CNKI), Wanfang Database, VIP Database, Chinese Biomedical Literature Database, PubMed, Web of Science, the Cochrane Library, and Embase. The search covered studies published from database inception to July 2026.

The search strategy combined Medical Subject Headings (MeSH) terms and free-text terms to ensure both the sensitivity and comprehensiveness of the search. The Chinese search terms mainly included “视觉艺术治疗(visual art therapy)”, “艺术治疗(art therapy)”, “老年人(older adults/elderly)”, “认知障碍(cognitive dysfunction)”, “记忆障碍(memory disorders)”, “注意力(attention)” and “执行功能(Executive functions)”. The corresponding English search terms included “visual art therapy”, “art therapy”, “aged”, “elderly”, “cognitive dysfunction”, “memory disorders”, “attention”, and “randomized controlled trial”. The detailed search strategies for each database are provided in the Supplementary Materials.

### 2.3 Inclusion and exclusion criteria

Inclusion criteria:

1. Participants: Older adults aged ≥60 years and older. No restrictions were placed on medication use or other participant characteristics.
2. Intervention type: The experimental group received interventions centered on visual art creation, including painting, drawing, collage, sculpture, calligraphy, and handicrafts (e.g., weaving and pottery). All intervention activities were delivered under the guidance of professional therapists. Interventions are pre-classified into the following two categories: 1) visual art therapy: programs using visual art creation as the sole primary modality; 2) combined art therapy: programs that retained visual art creation as the core while incorporating adjunct elements such as background music, brief warm-up activities, or artwork discussions.
3. Control conditions: Control groups received usual care (including assistance with activities of daily living, basic medical care, recreational activities, and environmental cleaning), standard cognitive rehabilitation (10 min of finger exercises, 30 min of core cognitive strategy training, and a 5-min summary session), online health education lectures (covering the epidemiology, assessment and diagnosis, prevention and treatment, functional training, and care of dementia), or no intervention.
4. Study design: Randomized controlled trials (RCTs).
5. Language: Only studies published in Chinese or English were included.

Exclusion criteria:

1. Studies with duplicate publication or overlapping data.
2. Studies classified as review articles, systematic reviews, study protocols, theoretical discussions, case reports, or conference papers.
3. Studies involving only non-visual art therapies, such as music therapy or dance therapy.

Outcome measures:

The primary outcome was global cognitive function, assessed using the Mini-Mental State Examination (MMSE), the Montreal Cognitive Assessment (MoCA), and the Standardized Mini-Mental Test (MMT). Secondary outcomes included activities of daily living (combining the Activities of Daily Living Scale [ADL] and the Chinese version of the Activities of Daily Living Scale [CAVDL], both scales follow the scoring criterion that “a lower score indicates better functional ability, while a higher score indicates poorer functional ability.”), the Verbal Fluency Test (VFT), and the Boston Naming Test (BNT).

### 2.4 Data extraction

The search results were imported into EndNote 21 for duplicate removal. Titles and abstracts were initially screened according to the predefined inclusion and exclusion criteria to exclude studies that clearly did not meet the eligibility criteria. The remaining articles were then reviewed in full text to determine final eligibility.

Data extraction was performed independently by two reviewers using a standardized form to collect the basic characteristics of each study, including author, publication year, study design, participant characteristics, intervention measures, control conditions, and outcome indicators. Any discrepancies were resolved through discussion with a third reviewer. All data extraction procedures were completed before the formal data synthesis to ensure the accuracy and completeness of the extracted data.

### 2.5 Risk of bias assessment

The Cochrane Risk of Bias tool was used to assess the methodological quality of the included randomized controlled trials (RCTs). The assessment domains included random sequence generation, allocation concealment, blinding, incomplete outcome data, selective reporting, and other potential sources of bias.

Each study was assessed independently by two reviewers, and any disagreements were resolved through discussion with a third reviewer. The final assessment results were classified as “low risk”, “high risk” or “unclear risk” and were reported in the systematic review. Subsequently, we evaluated the overall certainty of the evidence using the GRADE approach.

### 2.6 Data synthesis and statistical analysis

Meta-analysis was conducted using RevMan 5.4 and Stata 15.0. For continuous outcomes, pooled effect sizes were calculated as standardized mean differences (SMDs) or mean differences (MDs) with corresponding 95% confidence intervals (95% CIs) using the inverse-variance method. The SMD was used when outcomes were measured using different scales or units, whereas the MD was used when outcomes were measured using the same scale or unit. For studies reporting quartile data, the median and interquartile range (IQR) were converted into the mean and standard deviation for pooled analysis. Statistical heterogeneity was assessed using the χ² test and the I² statistic. If I² > 50% or P < 0.10, a random-effects model was applied; otherwise, a fixed-effects model was used.

For the primary outcome of global cognitive function, data from the Mini-Mental State Examination (MMSE), the Montreal Cognitive Assessment (MoCA), and the Standardized Mini-Mental Test (MMT) were analyzed. If more than one cognitive assessment was reported in the same study, MMSE data were preferentially used for pooled analysis. When the number of included studies was ≥10, funnel plots were constructed and Egger’s regression test was performed to assess potential publication bias.

## 3 Results

### 3.1 Study selection

**Figure 1:**
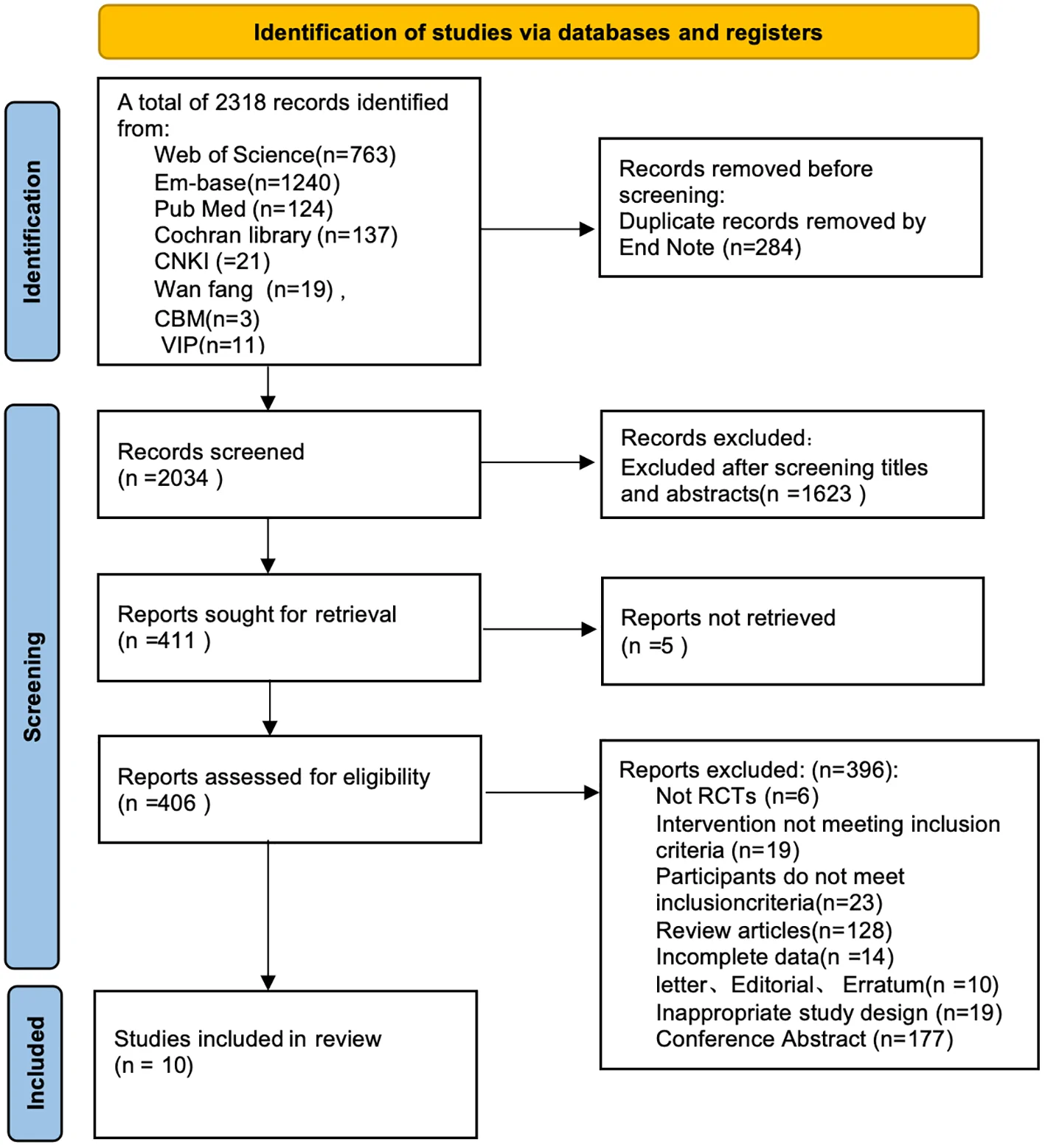
PRISMA flow diagram of the study selection process.

After the screening process, a total of 10 studies published in Chinese and English were ultimately included, involving 628 participants, of whom 318 were in the intervention group and 310 were in the control group. The study selection process is shown in **Figure 1**, and the basic characteristics of the included studies are summarized in **Table 1**.

**Table 1:** Characteristics of the included studies.

Source	Group size/age		Intervention description		Population	Intervention environment	Treatment frequency (sessions)	Session duration (min)	Outcomes
	IG	CG	IG	CG					
Timothy CY Kwok 2011 (20)	14 85.79 ± 4.93	17 85.76 ± 6.93	Visual Art Therapy	usual care	MCI	Nursing Homes	40	30	MMSE
ShuYu Tai 2016 (21)	14 70.21 ± 7.90	10 76.3 ± 7.07	Combined Art Therapy	maintaining daily activities	Mild AD	Community	12	180	MMSE
Eliana C. Ciasca 2018 (22)	31 66.1 ± 5.7	25 69.8 ± 6.4	Visual Art Therapy	usual care	depression	Hospital	20	90	MMSE
Junyu Zhao 2018 (23)	48 70.6 ± 6.9	45 69.5 ± 6.7	Combined Art Therapy	standard cognitive rehabilitation	MCI	Hospital	25	55-65	MoCA CVADL
Funda Çetinkaya 2019 (24)	15 74.5 ± 9	15 74.5 ± 9	Visual Art Therapy	usual care	healthy older adults	Nursing Homes	16	30-35	MMSE
Ran Duo 2021 (25)	29 82.27 ± 5.41	29 84.37 ± 6.94	Visual Art Therapy	usual care	MCI	Nursing Homes	20	60	MoCA ADL
Yuanjiao Yan 2022 (26)	72 72.32 ± 6.08	72 70.75 ± 5.55	Combined Art Therapy	online health education lectures on dementia	Mixed	Community、Hospital	24	60-90	MMSE MoCA VFT
Chenshan Huang 2025 (27)	40 87.5 ± 1.4	40 85 ± 2.36	Visual Art Therapy	usual care	MCI	Nursing Homes	28	60	MoCA ADL VFT BNT
Özge DEMIREL 2025 (28)	21 72.67 ± 7.32	21 73.81 ± 8.87	Visual Art Therapy	usual care	Mixed	Nursing Homes	8	40	MMT
Yuanjiao Yan 2025 (29)	34 70.8 ± 6.2	36 70.2 ± 5.4	Combined Art Therapy	usual care	Mixed	Community	24	90	MMSE MoCA VFT BNT

IG, intervention group; CG, control group; MCI, mild cognitive impairment; AD, Alzheimer’s disease; MMSE, Mini-Mental State Examination; MoCA, Montreal Cognitive Assessment; VFT, Verbal Fluency Test; BNT, Boston Naming Test; ADL, activities of daily living; CVADL, Chinese version of the Activities of Daily Living scale; MMT, Standardized Mini-Mental Test.

Mixed indicates participants with mixed cognitive conditions (e.g., MCI and mild Alzheimer’s disease). Combined Art Therapy: On the basis of core visual art creation, additional components such as background music, warm-up dance, and artwork discussion are included.

### 3.2 Risk of bias assessment

**Figure 2:**
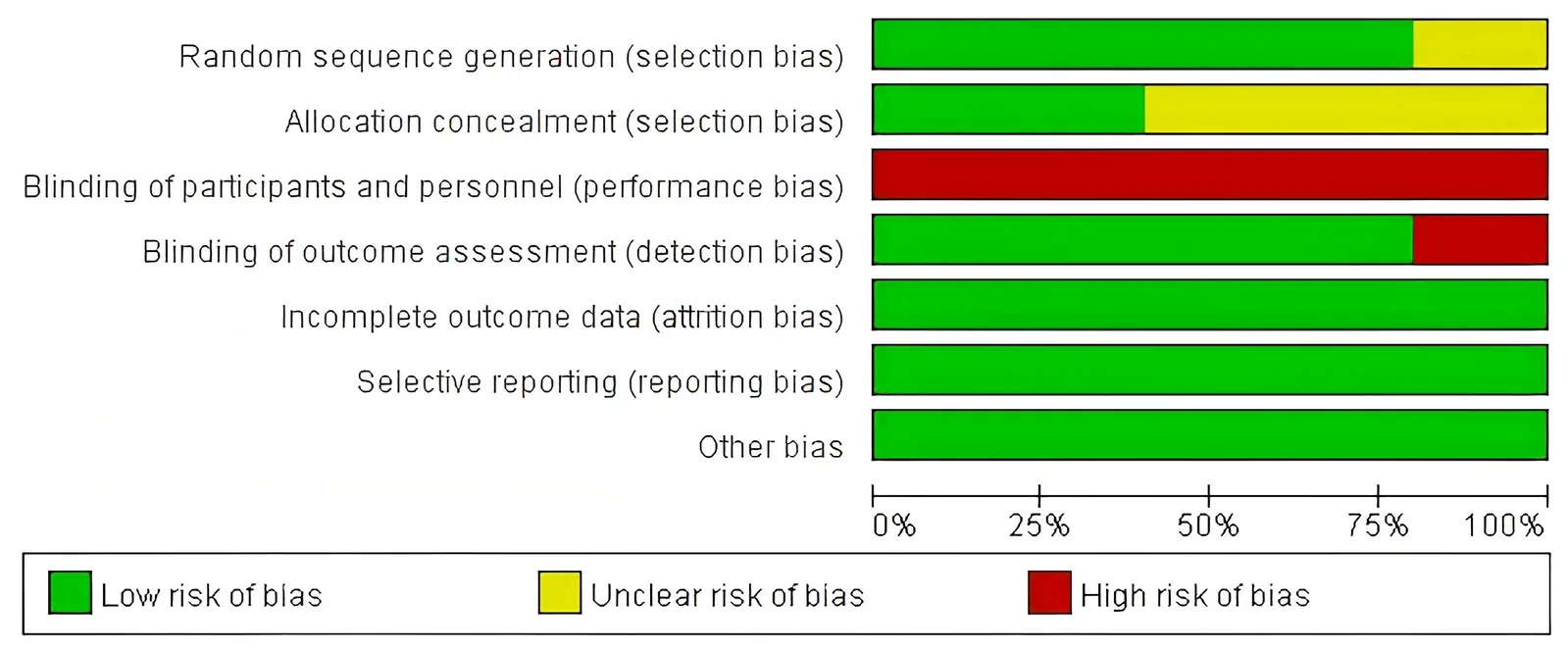
Risk of bias summary of the included studies.

**Figure 3:**
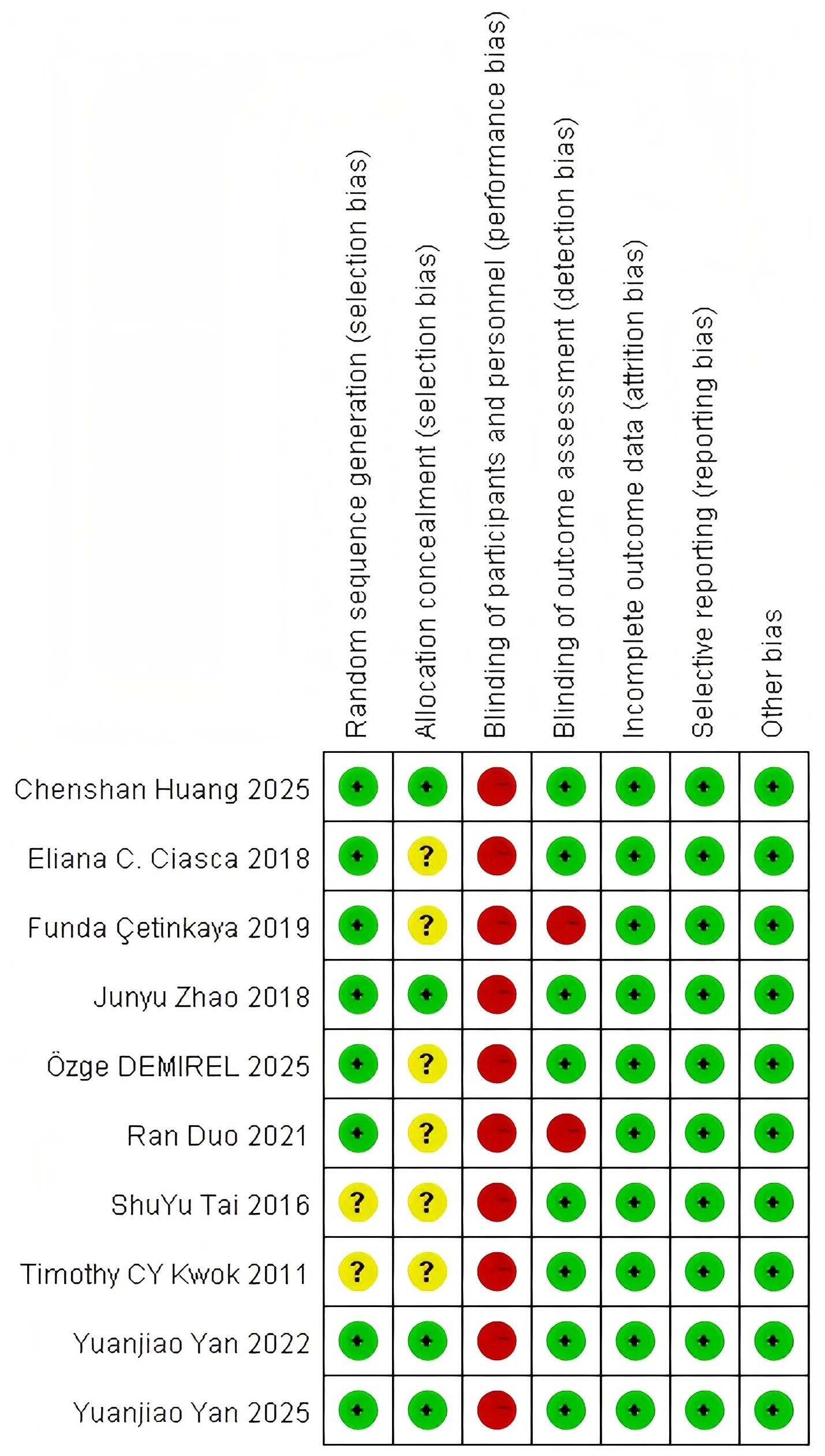
Risk of bias graph of the included studies.

We assessed the 10 included studies using the Cochrane Risk of Bias tool (RoB 1) (**Figures 2**, **3**). Eight studies (22–29) explicitly reported appropriate randomization methods (e.g., random number tables, computer-generated sequences); two studies (20, 21) did not provide sufficient information on sequence generation and were rated unclear risk. Allocation concealment was judged low risk in four studies (23, 26, 27, 29), and unclear risk in the remaining six (20–22, 24, 25, 28) due to insufficient reporting. Given the nature of visual art therapy, blinding of participants and personnel was generally infeasible; thus, all studies were rated high risk for performance bias. Outcome assessor blinding was rated low risk in eight studies (20–23, 26–29) and high risk in two (24, 25). All studies were judged low risk for incomplete outcome data, selective reporting, and other biases. Overall, methodological quality was acceptable; however, limitations in blinding and allocation concealment may introduce selection, performance, and measurement biases and potentially inflate effect estimates, thereby affecting the credibility and stability of the pooled results; findings should be interpreted with caution.

Certainty of evidence was appraised using GRADE. The overall certainty for global cognitive function was moderate; the certainty for the Verbal Fluency Test (VFT) and Boston Naming Test (BNT) was low; and the certainty for activities of daily living was very low. Detailed GRADE tables are provided in Supplementary Material S2.

### 3.3 Meta-analysis results

#### 3.3.1 Effects of visual art therapy on global cognitive function in older adults

**Figure 4:**
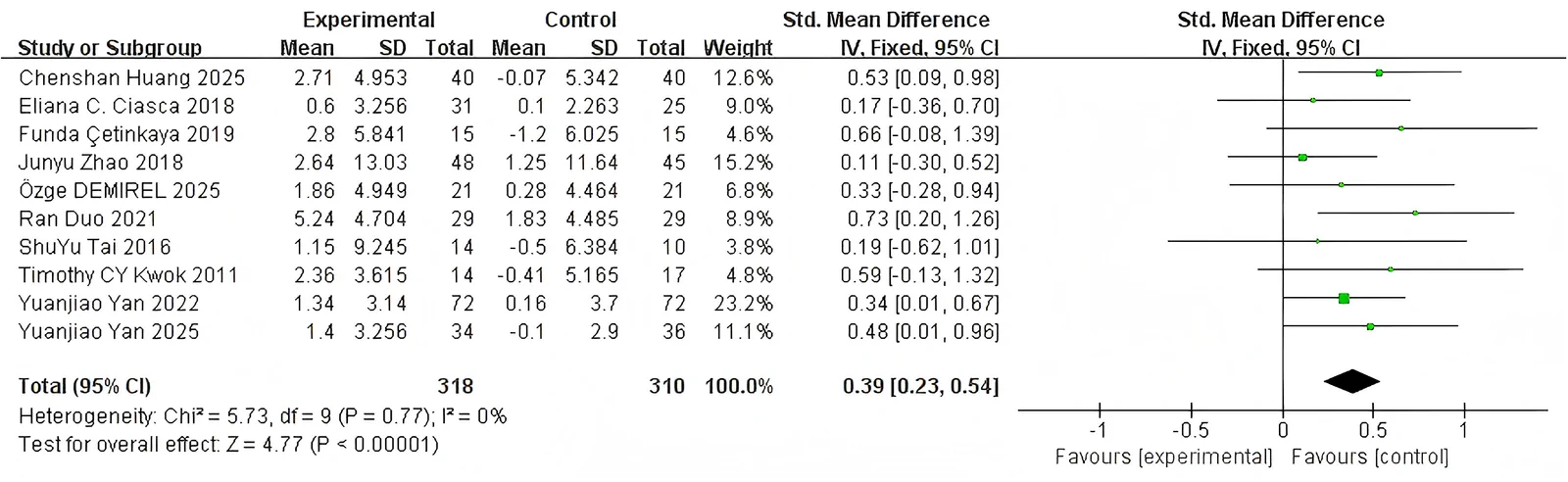
Forest plot of the effects of visual art therapy on global cognitive function in older adults.

A total of 10 studies were included to evaluate the effects of visual art therapy on cognitive function in older adults. The heterogeneity test indicated very low heterogeneity across studies (χ² = 5.73, P = 0.77, I² = 0%); therefore, a fixed-effects model was used. The meta-analysis showed that visual art therapy significantly improved global cognitive function in older adults, with a pooled effect size of SMD = 0.39 (95% CI: 0.23 to 0.54) (**Figure 4**). The overall effect test showed a statistically significant difference (Z = 4.77, P < 0.00001).

**Figure 5:**
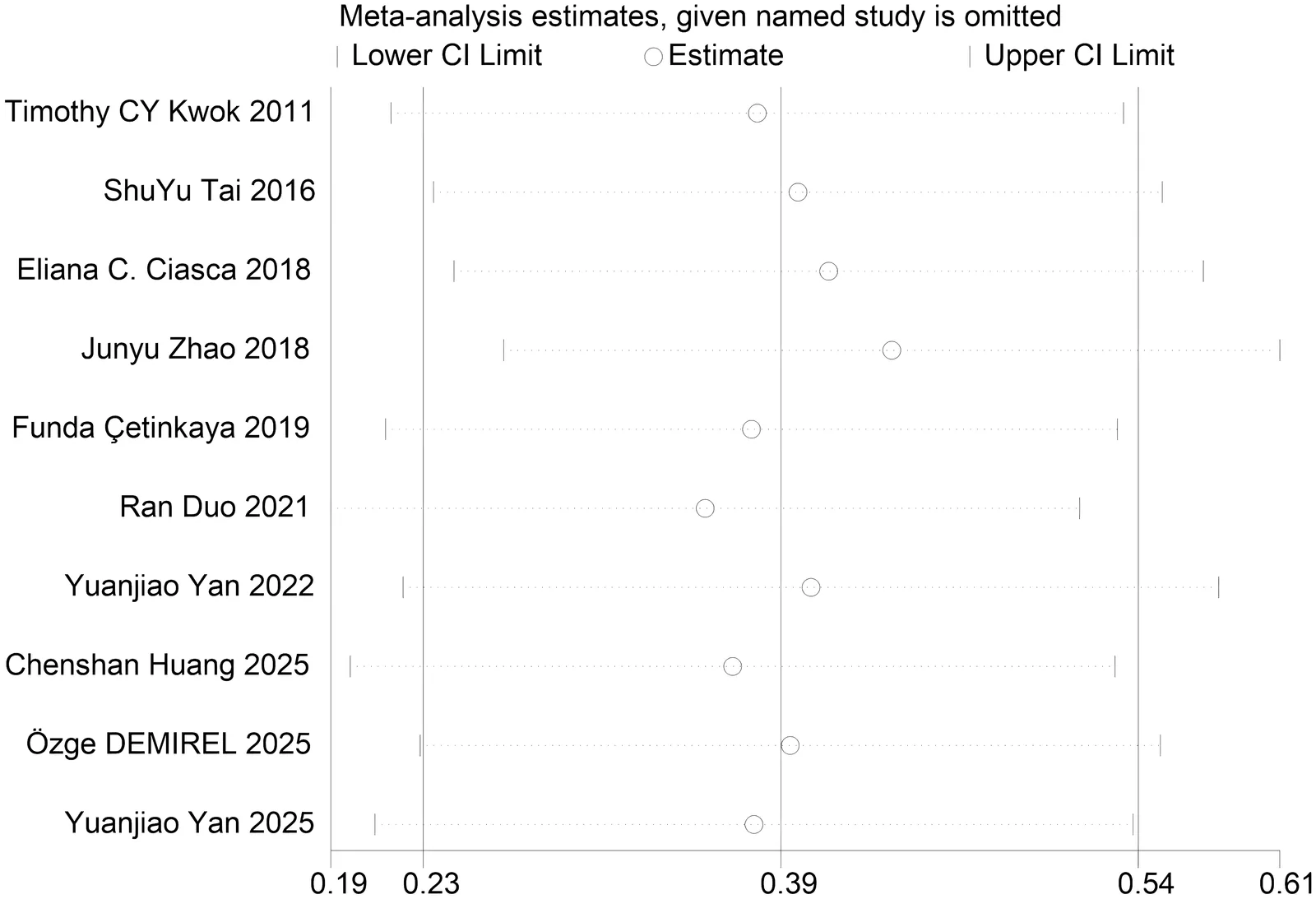
Sensitivity analysis of the effects of visual art therapy on global cognitive function in older adults.

**Figure 6:**
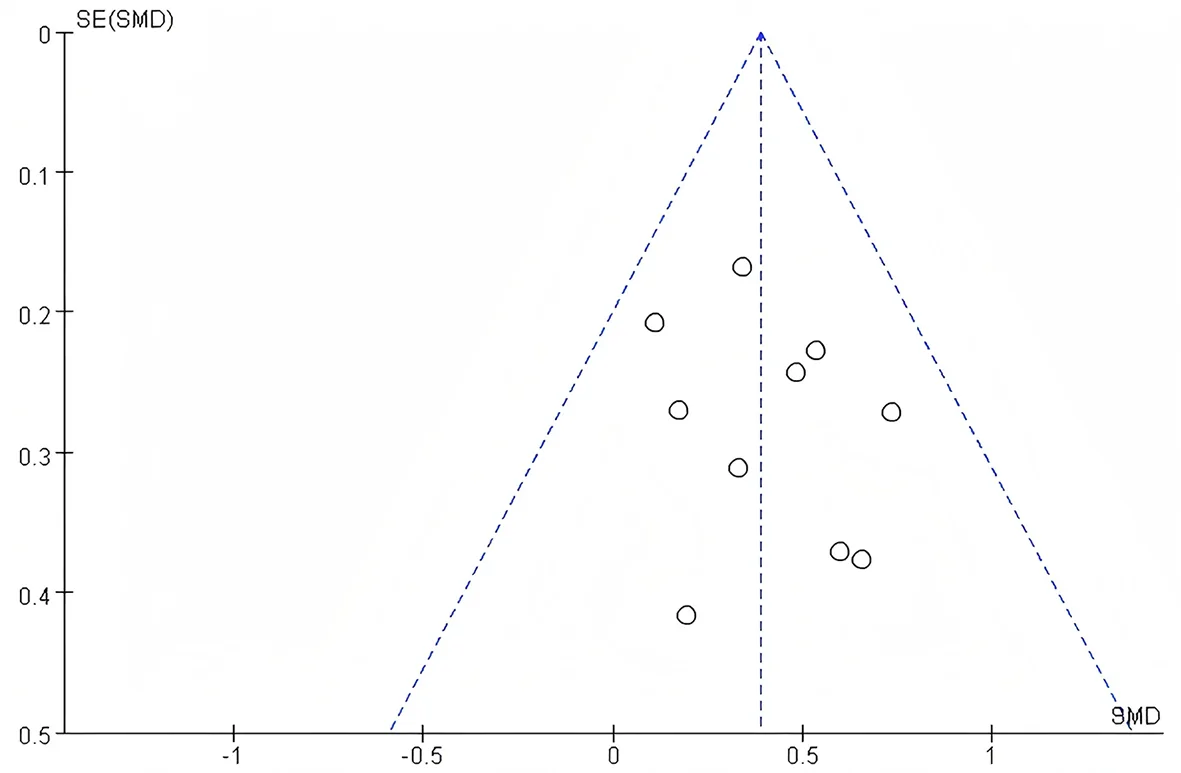
Funnel plot of the effects of visual art therapy on global cognitive function in older adults.

Sensitivity analysis showed that, after sequential removal of each study, the pooled effect size remained stable, and the direction and statistical significance of the results did not materially change, indicating that the findings were robust (**Figure 5**). The funnel plot showed a symmetrical distribution of data points, suggesting no obvious publication bias (**Figure 6**). Egger’s regression test also indicated no significant small-study effects (t = 0.83, P = 0.429).

#### 3.3.2 Effects of visual art therapy on language function in older adults

**Figure 7:**
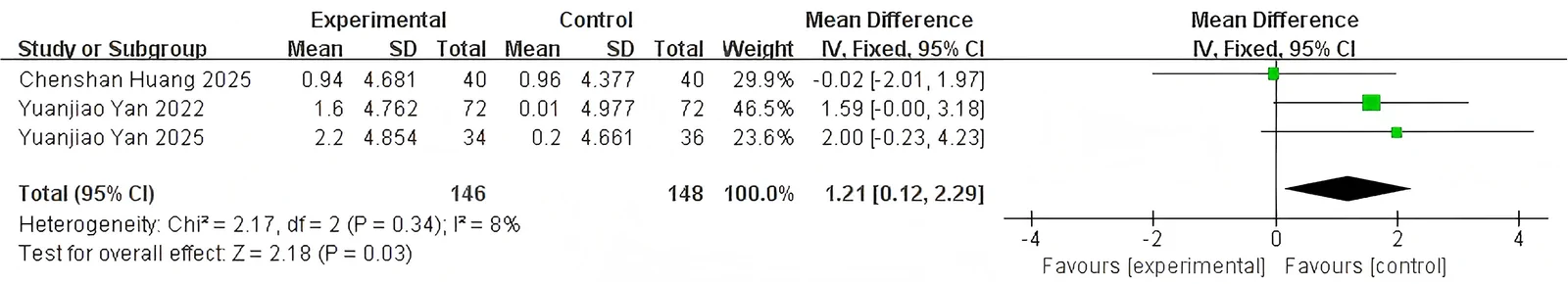
Forest plot of the effects of visual art therapy on verbal fluency (VFT) in older adults.

Three studies were included to evaluate the effects of visual art therapy on verbal fluency in older adults. The heterogeneity test showed low heterogeneity across studies (tu = 2.17, df = 2, P = 0.34, I3 = 8%); therefore, a fixed-effects model was applied. The meta-analysis showed that visual art therapy significantly improved verbal fluency in older adults, with a pooled effect size of MD = 1.21 (95% CI: 0.12 to 2.29). The overall effect test showed a statistically significant difference (Z = 2.18, P = 0.03) (**Figure 7**).

**Figure 8:**
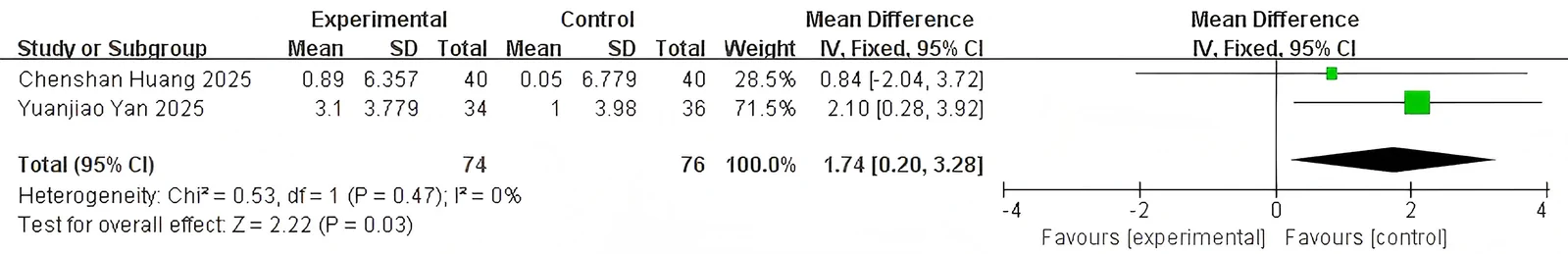
Forest plot of the effects of visual art therapy on naming ability (BNT) in older adults.

Two studies were included to evaluate the effects of visual art therapy on naming ability in older adults. The heterogeneity test indicated low heterogeneity across studies (tu = 0.53, df = 1, P = 0.47, I4 = 0%); therefore, a fixed-effects model was used. The meta-analysis showed that visual art therapy significantly improved naming ability in older adults, with a pooled effect size of MD = 1.74 (95% CI: 0.20 to 3.28). The overall effect test showed a statistically significant difference (Z = 2.22, P = 0.03) (**Figure 8**).

#### 3.3.3 Effects of visual art therapy on activities of daily living in older adults

Three studies were included to evaluate the effects of visual art therapy on activities of daily living in older adults. The heterogeneity test showed low heterogeneity across studies (tu = 0.58, df = 2, P = 0.75, I7 = 0%); therefore, a fixed-effects model was used.

**Figure 9:**
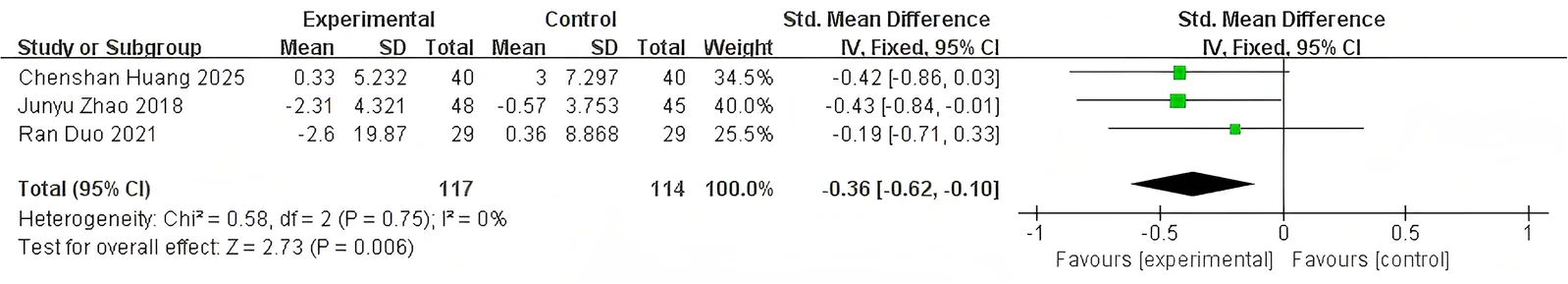
Forest plot of the effects of visual art therapy on activities of daily living in older adults.

The meta-analysis showed that visual art therapy significantly improved activities of daily living in older adults, with a pooled effect size of SMD = −0.36 (95% CI: −0.62 to −0.10). The overall effect test showed a statistically significant difference (Z = 2.73, P = 0.006) (**Figure 9**).

### 3.4 Subgroup analysis

To explore the influence of different intervention characteristics on the effects of visual art therapy on global cognitive function in older adults, subgroup analyses were conducted according to treatment frequency, treatment duration per session, intervention type, intervention setting, and participant characteristics (**Table 2**). Regarding treatment frequency, studies with es2 sessions did not show a significant improvement in cognitive function (SMD = 0.28, 95% CI: −0.21 to 0.77, P = 0.26), and low heterogeneity across studies (Iu = 0%). In contrast, studies with 13–24 sessions showed a significant intervention effect (SMD = 0.43, 95% CI: 0.22 to 0.64, P < 0.0001), with low heterogeneity across studies (Iu = 0%).

**Table 2:** Subgroup analysis of intervention characteristics on cognitive function.

Subgroups	No. of studies	Total sample (I+C)	Heterogeneity		SMD (95% CI)	Meta-analysis results	
			I^2^ (%)	P-value		Z	P
Treatment frequency (sessions)							
≤12	2	66	0	0.8	0.28 [-0.21,0.77]	1.13	0.26
13-24	5	358	0	0.59	0.43 [0.22, 0.64]	3.99	< 0.0001
≥25	3	204	17	0.3	0.36 [0.04, 0.67]	2.24	0.03
Session duration (min)							
30-40	3	103	0	0.76	0.50 [0.11, 0.90]	2.49	0.01
40-60	3	231	47	0.15	0.41 [0.15,0.67]	3.05	0.002
>60	4	294	0	0.84	0.33 [0.10, 0.56]	2.81	0.005
Type of intervention							
Combined Art Therapy	4	331	0	0.68	0.29 [0.08, 0.51]	2.66	0.008
Visual Art Therapy	6	297	0	0.74	0.49 [0.26, 0.72]	4.14	<0.0001
Intervention environment							
Community	3	238	0	0.81	0.37 [0.11, 0.62]	2.81	0.005
Nursing Homes	5	241	0	0.9	0.57 [0.31, 0.82]	4.3	< 0.0001
Population							
MCI	4	262	25	0.26	0.43 [0.18, 0.67]	3.41	0.0006
Mixed	3	256	0	0.88	0.38 [0.13, 0.63]	2.99	0.003

Regarding treatment duration per session, all three subgroups showed significant improvements; however, the effect size tended to decrease as the duration increased. The 30–40 min subgroup showed an SMD of 0.50 (95% CI: 0.11 to 0.90, P = 0.01), with no significant heterogeneity (It = 0%). The 40–60 min subgroup showed an SMD of 0.41 (95% CI: 0.15 to 0.67, P = 0.002), with moderate heterogeneity (It = 47%). The >60 min subgroup showed an SMD of 0.33 (95% CI: 0.10 to 0.56, P = 0.005), with no heterogeneity (I^2^ = 0%).

Regarding intervention type, both combined art therapy and visual art therapy alone significantly improved cognitive function. The pooled effect size for combined art therapy was SMD = 0.29 (95% CI: 0.08 to 0.51, P = 0.008; I² = 0%), whereas that for visual art therapy alone was SMD = 0.49 (95% CI: 0.26 to 0.72, P <0.0001; I^2^ = 0%).

Regarding intervention setting, visual art therapy significantly improved cognitive function in both community and nursing home settings. The effect was more pronounced among nursing home residents (SMD = 0.57, 95% CI: 0.31 to 0.82, P < 0.0001; I. = 0%) than among community-dwelling older adults (SMD = 0.37, 95% CI: 0.11 to 0.62, P = 0.005; I^2^ = 0%).

Regarding participant characteristics, significant improvements were observed in both participants with mild cognitive impairment (MCI) (SMD = 0.43, 95% CI: 0.18 to 0.67, P = 0.0006; I0 = 25%) and mixed populations (SMD = 0.38, 95% CI: 0.13 to 0.63, P = 0.003; I0 = 0%).

Overall, the subgroup analyses further support the positive and stable effects of visual art therapy on cognitive function in older adults under different intervention conditions.

## 4 Discussion

This systematic review and meta-analysis included 10 randomized controlled trials involving 628 older adults and systematically evaluated the effects of visual art therapy on cognitive function in this population. The results showed that visual art therapy significantly improves global cognitive function and demonstrated advantages over control conditions in language function (VFT and BNT) as well as activities of daily living.

### 4.1 Effects of visual art therapy on global cognitive function in older adults and influencing factors

Previous studies have suggested that visual art therapy may influence cognitive function through mechanisms related to cognitive reserve and neuroplasticity (30). During the creative process of visual art therapy, individuals are required to plan and organize artistic tasks, integrate visual and semantic information, dynamically adjust actions during task execution, and reflect on and verbally interpret their completed work (31). This process may simultaneously engages multiple cognitive domains, including orientation, attentional control, visuospatial processing, language ability, and abstract reasoning (32). Neuroimaging studies have shown that artistic creation tasks can significantly activate the dorsal visual stream, prefrontal cortex, and parietal regions. Among these regions, the prefrontal cortex primarily supports artistic conception and decision-making, whereas the parietal regions integrate fine motor control with higher-order cognitive processes (33, 34). Through repeated engagement in complex tasks, it has been hypothesized that the coordinated activation of these regions may promote neuroplastic changes and strengthen functional connectivity, thereby enhancing cognitive reserve (35, 36). It should be noted that these neurobiological explanations are primarily based on basic research and theoretical inferences from related fields, rather than direct evidence from the clinical trials included in this review. The core findings of this study remain based on effect sizes reflecting improvements in clinical symptoms.

Consistent with these mechanisms, the present meta-analysis shows that visual art therapy significantly improved global cognitive function in older adults (SMD = 0.39, P < 0.00001), which is in line with previous systematic reviews and meta-analyses (37, 38). Although this meta-analysis showed low statistical heterogeneity (It = 0%), the 10 included studies still differed in intervention duration, number of sessions, and participant characteristics; statistical homogeneity does not preclude meaningful clinical heterogeneity. Subgroup analyses were further conducted to provide evidence for optimizing intervention strategies in clinical practice.

Comparing different intervention doses, 13–24 sessions showed a relatively more stable effect trend on global cognitive function(SMD =0.43, P< 0.0001), suggesting that visual art therapy may require a certain intervention cycle to accumulate benefits. As a non-pharmacological intervention, its efficacy typically builds gradually through repeated stimulation, and very short courses may be insufficient to produce sustained improvement (39).

Regarding single-session duration, all time settings showed some improvement, but effect sizes did not increase monotonically with longer durations. Among the interventions included, sessions of 30–40 minutes yielded a relatively larger effect size (SMD = 0.50, P = 0.01), which may reflect that a moderate duration helps maintain attention and creative engagement in older adults (40), whereas excessively long sessions may lead to fatigue and reduced motivation, thereby weakening the intervention effect. Given the limited number of studies per subgroup, these findings are exploratory and should be interpreted with caution.

Regarding intervention type, both combined art-based interventions and visual art therapy alone significantly improved global cognitive function in older adults (combined art therapy: SMD = 0.29, P = 0.008; visual art therapy: SMD = 0.49, P<0.0001). Visual art therapy mainly emphasizes spatial construction and visual memory (41), with the creative process requires sustained visuospatial processing and fine motor coordination. Combined art therapy incorporate additional components such as background music and warm-up dance, which may generate broader activation across multiple cognitive domains (42–44). It should be noted that the above numerical differences should be interpreted as exploratory findings, and future well-designed studies are needed to directly compare the relative effectiveness of the two intervention modalities.

Regarding intervention setting, subgroup analysis indicated that visual art therapy significantly improved cognitive function in both community and nursing home settings, with a relatively larger effect size observed among nursing home residents (SMD = 0.57, P < 0.0001). Previous studies have suggested that older adults living in nursing homes often experience reduced social interaction and limited environmental stimulation, which may make them more responsive to structured and group-based art interventions (45, 46). Therefore, implementing regular art activities in nursing homes may be particularly beneficial for maintaining and improving cognitive function. Given the limited number of included studies, these findings are exploratory and should be interpreted with caution.

Regarding participant characteristics, older adults with different cognitive statuses may benefit from visual art therapy, with a relatively larger effect size observed among those with mild cognitive impairment (MCI) (SMD = 0.43, P = 0.0006). This finding is consistent with previous research suggesting that individuals with cognitive decline may benefit more from cognitive training than cognitively healthy individuals or patients with dementia. Individuals in the early stage of cognitive decline may retain greater neuroplasticity, making their cognitive functions more responsive to intervention (37, 47).Given the limited number of included studies, these findings are exploratory and should be interpreted with caution.

### 4.2 Effects of visual art therapy on language function in older adults and influencing factors

Language function is an important component of higher-order cognitive function and encompasses both language comprehension and expression (48). Visual art therapy may influence language function through multiple pathways (49). The artwork sharing and discussion stage following artistic creation provides older adults with opportunities for verbal expression and social interaction. The processes of describing, interpreting, and discussing artworks in individual or group contexts require lexical retrieval, semantic organization, and narrative construction, thereby stimulating verbal fluency and naming ability (27). In addition, the semantic retrieval and narrative construction processes activated during artistic activities may engage language-related neural networks in the temporal and frontal lobes, which contribute to the maintenance of verbal fluency and naming ability (50).

The results of this meta-analysis showed that visual art therapy significantly improved verbal fluency, as measured by the Verbal Fluency Test (VFT) (MD = 1.21, P = 0.03), and naming ability, as measured by the Boston Naming Test (BNT) (MD = 1.74, P = 0.03), in older adults, indicating beneficial effects on language function. These findings are broadly consistent with the conclusions of previous systematic reviews (37, 51). Notably, the present study is among the first meta-analyses to examine two specific dimensions of language function verbal fluency as assessed by the Verbal Fluency Test and naming ability as assessed by the Boston Naming Test, thereby providing more comprehensive evidence on the effects of visual art therapy on language outcomes. During visual art therapy, participants are required to understand task instructions, retrieve relevant experiences, and, during the sharing phase, organize language to express their thoughts and emotions; these processes may jointly facilitate improvements in language function (27). However, the analyses of Verbal Fluency Test (three studies) and Boston Naming Test (two studies) were based on a limited number of studies, and there was heterogeneity in language assessment tools and intervention protocols across studies; therefore, these findings should be interpreted with caution.

### 4.3 Effects of visual art therapy on activities of daily living in older adults and influencing factors

Activities of daily living are an important indicator for evaluating the functional status and quality of life of older adults (52). Visual art therapy, which involves creative activities using mixed media such as painting, collage, and sculpture, may improve communication ability, fine motor control, hand-eye coordination, and task execution in older adults, thereby enabling them to complete daily activities more independently (53, 54). Furthermore, these activities require older adults to engage in complex tasks, including information detection, identification, discrimination, spatial localization, and attentional shifting across multiple tasks (55). Improvements in attention, cognitive function, and emotional status resulting from therapeutic activities may indirectly promote the maintenance of independence in daily living among older adults (56).

The results of the present meta-analysis showed that visual art therapy significantly improved activities of daily living in older adults (SMD = −0.36, P = 0.006), indicating a beneficial role in maintaining or improving functional status. This finding is consistent with previous empirical studies (57, 58). During art therapy sessions, participants are required to complete creative tasks ranging from simple to complex, involving planning, aesthetic engagement, organized expression, and task execution. These processes may contribute to the maintenance of functional abilities related to daily life. Given the limited number of included studies, these findings are exploratory and should be interpreted with caution.

## 5 Conclusions

This study synthesized evidence from 10 randomized controlled trials through a systematic review and meta-analysis. The results indicate that visual art therapy can significantly improve global cognitive function in older adults and may also have beneficial effects on language function and activities of daily living. Subgroup analyses further suggest that the effectiveness of visual art therapy may be influenced by multiple factors. In terms of intervention dose, an overall course of 13–24 sessions associate an intervention cycle with relatively larger effect sizes, and sustained intervention may help maintain the improvement trend. Regarding session duration, 30–40 min per session appears to be appropriate, whereas excessively long sessions may reduce intervention effectiveness. In terms of intervention format, both combined art therapy and visual art therapy alone demonstrated significant improvements in global cognitive function in older adults. Regarding intervention settings and participant characteristics, cognitive improvements appeared to be more pronounced among older adults receiving interventions in nursing home settings, and those with mild cognitive impairment appeared to benefit more than mixed populations, suggesting that early intervention may have potential value.

However, several limitations should be acknowledged. First, the number of studies included for the secondary outcomes was limited; therefore, these findings should be interpreted with caution. Second, some studies did not provide sufficient information on allocation concealment and blinding procedures, which may have introduced potential bias and affected the precision of the effect estimates. Third, because of the limited number of included studies and the availability of original data, further subgroup analyses of specific intervention components, such as the type of visual art therapy, the level of therapist involvement, and the degree of creative freedom, could not be performed, precluding examination of potential moderators and their interactions on cognitive outcomes. Accordingly, although the evidence provides preliminary support for benefits of visual art therapy on cognitive function in older adults, it remains insufficient to underpin definitive clinical recommendations. Future investigations should employ larger samples, extended follow-up, and rigorously designed randomized controlled trials to confirm efficacy and to delineate optimal intervention parameters and target populations.

## Data Availability

The original contributions presented in the study are included in the article/**Supplementary Material**. Further inquiries can be directed to the corresponding author.
