# Supplementary material for: Effects of visual art therapy on cognitive function in older adults: a systematic review and meta-analysis
Source: Front Psychiatry. 2026 Aug 19;17:1828257. doi: 10.3389/fpsyt.2026.1828257 (PMC13534113; doi:10.3389/fpsyt.2026.1828257)
Supplement: Supplementary file 1 [file Table1.pdf]

Table S1 The search strategy		
**Datebases**	**Searching strategy**	**Literature number**
**Pubmed**	((((((((((((Art Therapy[MeSH Terms]) OR (Art Therapy[Title/Abstract])) OR (Therapy, Art[Title/Abstract])) OR (Art Therapies[Title/Abstract])) OR (Therapies, Art[Title/Abstract])) OR (Visual Intervention[Title/Abstract])) OR (Drawing Intervention[Title/Abstract])) OR (Drawing Therapy[Title/Abstract])) OR (painting Therapy[Title/Abstract])) OR (Visual art Therapy[Title/Abstract])) OR ((((((((((((Art[MeSH Terms]) OR (Art[Title/Abstract])) OR (Collage making[Title/Abstract])) OR (creative arts[Title/Abstract])) OR (Draw[Title/Abstract])) OR (Sketching[Title/Abstract])) OR (Calligraphic[Title/Abstract])) OR (Artistic pottery[Title/Abstract])) OR (Sculpting[Title/Abstract])) OR (Craft[Title/Abstract])) OR (Colouring[Title/Abstract])) OR (Sandpainting[Title/Abstract]))) AND (((Aged[MeSH Terms]) OR (Aged[Title/Abstract])) OR (Elderly[Title/Abstract]))) AND (((((((((((((Cognition[MeSH Terms]) OR (Cognition[Title/Abstract])) OR (Cognitions[Title/Abstract])) OR (Cognitive Function[Title/Abstract])) OR (Cognitive Functions[Title/Abstract])) OR (Function, Cognitive[Title/Abstract])) OR (Functions, Cognitive[Title/Abstract])) OR (Insight[Title/Abstract])) OR (Insights[Title/Abstract])) OR (((((((((((((((((((((((((((Cognitive Dysfunction[MeSH Terms]) OR (Cognitive Dysfunction[Title/Abstract])) OR (Cognitive Dysfunctions[Title/Abstract])) OR (Dysfunction, Cognitive[Title/Abstract])) OR (Dysfunctions, Cognitive[Title/Abstract])) OR (Cognitive Disorder[Title/Abstract])) OR (Cognitive Disorders[Title/Abstract])) OR (Disorder, Cognitive[Title/Abstract])) OR (Disorders, Cognitive[Title/Abstract])) OR (Cognitive Impairments[Title/Abstract])) OR (Cognitive Impairment[Title/Abstract])) OR (Impairment, Cognitive[Title/Abstract])) OR (Impairments, Cognitive[Title/Abstract])) OR (Mild Cognitive Impairment[Title/Abstract])) OR (Cognitive Impairment, Mild[Title/Abstract])) OR (Cognitive Impairments,	124

	Mild[Title/Abstract])) OR (Impairment, Mild Cognitive[Title/Abstract])) OR (Impairments, Mild Cognitive[Title/Abstract])) OR (Mild Cognitive Impairments[Title/Abstract])) OR (Cognitive Decline[Title/Abstract])) OR (Cognitive Declines[Title/Abstract])) OR (Decline, Cognitive[Title/Abstract])) OR (Declines, Cognitive[Title/Abstract])) OR (Mental Deterioration[Title/Abstract])) OR (Deterioration, Mental[Title/Abstract])) OR (Deteriorations, Mental[Title/Abstract])) OR (Mental Deteriorations[Title/Abstract]))) OR (((((((((((((((((((((((((((Memory Disorders[MeSH Terms]) OR (Memory Disorders[Title/Abstract])) OR (Memory Disorder[Title/Abstract])) OR (Retention Disorders, Cognitive[Title/Abstract])) OR (Cognitive Retention Disorder[Title/Abstract])) OR (Cognitive Retention Disorders[Title/Abstract])) OR (Retention Disorder, Cognitive[Title/Abstract])) OR (Memory Loss[Title/Abstract])) OR (Memory Losses[Title/Abstract])) OR (Age-Related Memory Disorders[Title/Abstract])) OR (Age-Related Memory Disorder[Title/Abstract])) OR (Age Related Memory Disorders[Title/Abstract])) OR (Memory Disorder, Age-Related[Title/Abstract])) OR (Memory Disorders, Age-Related[Title/Abstract])) OR (Memory Disorders, Age Related[Title/Abstract])) OR (Memory Deficits[Title/Abstract])) OR (Deficit, Memory[Title/Abstract])) OR (Deficits, Memory[Title/Abstract])) OR (Memory Deficit[Title/Abstract])) OR (Memory Disorder, Semantic[Title/Abstract])) OR (Memory Disorders, Semantic[Title/Abstract])) OR (Semantic Memory Disorders[Title/Abstract])) OR (Semantic Memory Disorder[Title/Abstract])) OR (Memory Disorder, Spatial[Title/Abstract])) OR (Memory Disorders, Spatial[Title/Abstract])) OR (Spatial Memory Disorders[Title/Abstract])) OR (Spatial Memory Disorder[Title/Abstract]))) OR ((((((((((Attention[MeSH Terms]) OR (Attention[Title/Abstract])) OR (Focus of Attention[Title/Abstract])) OR (Attention Focus[Title/Abstract])) OR (Mental Concentration[Title/Abstract])) OR (Concentration, Mental[Title/Abstract])) OR (Social Attention[Title/Abstract])) OR (Attention, Social[Title/Abstract])) OR (Selective Attention[Title/Abstract])) OR (Attention, Selective[Title/Abstract]))) OR (((((((Executive Function[MeSH Terms]) OR (Executive Function[Title/Abstract])) OR (Executive Functions[Title/Abstract])) OR (Function, Executive[Title/Abstract])) OR (Functions, Executive[Title/Abstract])) OR (Executive Control[Title/Abstract])) OR (Executive Controls[Title/Abstract])))) AND (((randomized controlled trial[MeSH Terms]) OR (randomized controlled trial[Title/Abstract])) OR (RCT[Title/Abstract]))	

**Web of science**	#1 TS=("art therapy" OR "therapy, art" OR "art therapies" OR "therapies, art" OR "visual intervention" OR "drawing intervention" OR "drawing therapy" OR "painting therapy" OR "visual art therapy" OR art OR "collage making" OR "creative arts" OR draw* OR sketching OR calligraph* OR "artistic pottery" OR sculpt* OR craft* OR colouring OR coloring OR sandpainting) 31474768<br><br>#2 TS=(aged OR elderly) 13470085<br><br>#3 TS=(cognition OR cognitions OR "cognitive function" OR "function, cognitive" OR "functions, cognitive" OR insight OR insights OR "cognitive dysfunction" OR "dysfunction, cognitive" OR "dysfunctions, cognitive" OR "cognitive disorder" OR "disorder, cognitive" OR "disorders, cognitive" OR "cognitive impairment" OR "impairment, cognitive" OR "impairments, cognitive" OR "mild cognitive impairment" OR "cognitive impairment, mild" OR "cognitive impairments, mild" OR "impairment, mild cognitive" OR "impairments, mild cognitive" OR "cognitive decline" OR "decline, cognitive" OR "declines, cognitive" OR "mental deterioration" OR "deterioration, mental" OR "deteriorations, mental" OR "mental deteriorations" OR "memory disorder" OR "retention disorders, cognitive" OR "cognitive retention disorder" OR "retention disorder, cognitive" OR "memory loss" OR "age-related memory disorder" OR "age related memory disorder" OR "memory disorder, age-related" OR "memory disorders, age-related" OR "memory disorders, age related" OR "memory deficit" OR "deficit, memory" OR "deficits, memory" OR "memory disorder, semantic" OR "memory disorders, semantic" OR "semantic memory disorder" OR "memory disorder, spatial" OR "memory disorders, spatial" OR "spatial memory disorder" OR attention OR "focus of attention" OR "attention focus" OR "mental concentration" OR "concentration, mental" OR "social attention" OR "attention, social" OR "selective attention" OR "attention, selective" OR "executive function" OR "function, executive" OR "functions, executive" OR "executive control") 6277952<br><br>#4 #1 AND #2 AND #3 68244<br><br>#5 TS=("randomized controlled trial" OR "randomised controlled trial") 352765	**763**

	#5AND #4 763	

<table>
<tr><td rowspan="9">Embase</td><td colspan="3">Embase session results (16 Jul 2026)</td><td rowspan="9">1240</td></tr>
<tr><td>No.</td><td>Query</td><td>Results</td></tr>
<tr><td>#9</td><td>#7 AND #8</td><td>1240</td></tr>
<tr><td>#8</td><td>'randomized controlled trial'/exp OR 'randomized controlled trial':ab,ti OR 'rct':ab,ti</td><td>1291335</td></tr>
<tr><td>#7</td><td>#1 AND #2 AND #6</td><td>11173</td></tr>
<tr><td>#6</td><td>#3 OR #4 OR #5</td><td>5809541</td></tr>
<tr><td>#5</td><td>'attention'/exp OR 'attention':ab,ti OR 'focus of attention':ab,ti OR 'attention focus':ab,ti OR 'mental concentration':ab,ti OR 'concentration, mental':ab,ti OR 'social attention':ab,ti OR 'attention, social':ab,ti OR 'selective attention':ab,ti OR 'attention, selective':ab,ti OR 'executive function'/exp OR 'executive function':ab,ti OR 'executive functions':ab,ti OR 'function, executive':ab,ti OR 'functions, executive':ab,ti OR 'executive control':ab,ti OR 'executive controls':ab,ti</td><td>1270762</td></tr>
<tr><td>#4</td><td>'memory disorders'/exp OR 'memory disorders':ab,ti OR 'memory disorder':ab,ti OR 'retention disorders, cognitive':ab,ti OR 'cognitive retention disorder':ab,ti OR 'cognitive retention disorders':ab,ti OR 'retention disorder, cognitive':ab,ti OR 'memory loss':ab,ti OR 'memory losses':ab,ti OR 'age-related memory disorders':ab,ti OR 'age-related memory disorder':ab,ti OR 'age related memory disorders':ab,ti OR 'memory disorder, age-related':ab,ti OR 'memory disorders, age-related':ab,ti OR 'memory disorders, age related':ab,ti OR 'memory deficits':ab,ti OR 'deficit, memory':ab,ti OR 'deficits, memory':ab,ti OR 'memory deficit':ab,ti OR 'memory disorder, semantic':ab,ti OR 'memory disorders, semantic':ab,ti OR 'semantic memory disorders':ab,ti OR 'semantic memory disorder':ab,ti OR 'memory disorder, spatial':ab,ti OR 'memory disorders, spatial':ab,ti OR 'spatial memory disorders':ab,ti OR 'spatial memory disorder':ab,ti</td><td>122081</td></tr>
</table>

#3	'cognition'/exp OR 'cognition':ab,ti OR 'cognitions':ab,ti OR 'cognitive function':ab,ti OR 'cognitive functions':ab,ti OR 'function, cognitive':ab,ti OR 'functions, cognitive':ab,ti OR 'insight':ab,ti OR 'insights':ab,ti OR 'cognitive dysfunction' OR 'cognitive dysfunction':ab,ti OR 'cognitive dysfunctions':ab,ti OR 'dysfunction, cognitive':ab,ti OR 'dysfunctions, cognitive':ab,ti OR 'cognitive disorder':ab,ti OR 'cognitive disorders':ab,ti OR 'disorder, cognitive':ab,ti OR 'disorders, cognitive':ab,ti OR 'cognitive impairments':ab,ti OR 'cognitive impairment':ab,ti OR 'impairment, cognitive':ab,ti OR 'impairments, cognitive':ab,ti OR 'mild cognitive impairment':ab,ti OR 'cognitive impairment, mild':ab,ti OR 'cognitive impairments, mild':ab,ti OR 'impairment, mild cognitive':ab,ti OR 'impairments, mild cognitive':ab,ti OR 'mild cognitive impairments':ab,ti OR 'cognitive decline':ab,ti OR 'cognitive declines':ab,ti OR 'decline, cognitive':ab,ti OR 'declines, cognitives':ab,ti OR 'mental deterioration':ab,ti OR 'deterioration, mental':ab,ti OR 'deteriorations, mental':ab,ti OR 'mental deteriorations':ab,ti	5209309
#2	'aged'/exp OR 'aged':ab,ti OR 'elderly':ab,ti	5844383
#1	'art therapy'/exp OR 'art therapy':ab,ti OR 'therapy, art':ab,ti OR 'art therapies':ab,ti OR 'therapies, art':ab,ti OR 'visual intervention':ab,ti OR 'drawing intervention':ab,ti OR 'drawing therapy':ab,ti OR 'painting therapy':ab,ti OR 'visual art therapy':ab,ti OR 'art'/exp OR ' art':ab,ti OR 'arts':ab,ti OR 'collage making':ab,ti OR 'creative arts':ab,ti OR 'draw':ab,ti OR 'sketching':ab,ti OR 'calligraphic':ab,ti OR 'artistic pottery':ab,ti OR 'sculpting':ab,ti OR 'craft':ab,ti OR 'colouring':ab,ti OR 'sandpainting':ab,ti	439712

Cochrane Library	D Search Hits<br><br>#1 (Art Therapy):ti,ab,kw OR (Therapy, Art):ti,ab,kw OR (Art Therapies):ti,ab,kw OR (Therapies, Art):ti,ab,kw OR (Visual Intervention):ti,ab,kw 56729<br><br>#2 (Drawing Intervention):ti,ab,kw OR (Drawing Therapy):ti,ab,kw OR (painting Therapy):ti,ab,kw OR (Visual art Therapy):ti,ab,kw OR (Art):ti,ab,kw 15160<br><br>#3 (Arts):ti,ab,kw OR (Collage making):ti,ab,kw OR (creative arts):ti,ab,kw OR (Draw):ti,ab,kw OR (Sketching):ti,ab,kw 8031<br><br>#4 (Calligraphic):ti,ab,kw OR (Artistic pottery):ti,ab,kw OR (Sculpting):ti,ab,kw OR (Craft):ti,ab,kw OR (colouring):ti,ab,kw 1762<br><br>#5 (Sandpainting):ti,ab,kw 0<br><br>#6 #1 OR #2 OR #3 OR #4 OR #5 72220<br><br>#7 (Aged):ti,ab,kw OR (Elderly):ti,ab,kw 772250<br><br>#8 (Cognition):ti,ab,kw OR (Cognitions):ti,ab,kw OR (Cognitive Function):ti,ab,kw OR (Cognitive Functions):ti,ab,kw OR (Function, Cognitive):ti,ab,kw 68473<br><br>#9 (Functions, Cognitive):ti,ab,kw OR (Insight):ti,ab,kw OR (Insights):ti,ab,kw 32678<br><br>#10 (Cognitive Dysfunction):ti,ab,kw OR (Cognitive Dysfunctions):ti,ab,kw OR (Dysfunction, Cognitive):ti,ab,kw OR (Dysfunctions, Cognitive):ti,ab,kw OR (Cognitive Disorder):ti,ab,kw 36339	**137**

	#11 (Cognitive Disorders):ti,ab,kw OR (Disorder, Cognitive):ti,ab,kw OR (Disorders, Cognitive):ti,ab,kw OR (Cognitive Impairments):ti,ab,kw OR (Cognitive Impairment):ti,ab,kw 60495<br><br>#12 (Impairment, Cognitive):ti,ab,kw OR (Impairments, Cognitive):ti,ab,kw OR (Mild Cognitive Impairment):ti,ab,kw OR (Cognitive Impairment, Mild):ti,ab,kw OR (Cognitive Impairments, Mild):ti,ab,kw 25371<br><br>#13 (Impairment, Mild Cognitive):ti,ab,kw OR (Impairments, Mild Cognitive):ti,ab,kw OR (Mild Cognitive Impairments):ti,ab,kw OR (Cognitive Decline):ti,ab,kw OR (Cognitive Declines):ti,ab,kw 15834<br><br>#14 (Decline, Cognitive):ti,ab,kw OR (Declines, Cognitive):ti,ab,kw OR (Mental Deterioration):ti,ab,kw OR (Deterioration, Mental):ti,ab,kw OR (Deteriorations, Mental):ti,ab,kw 10733<br><br>#15 (Mental Deteriorations):ti,ab,kw 25<br><br>#16 (Memory Disorders):ti,ab,kw OR (Memory Disorder):ti,ab,kw OR (Retention Disorders, Cognitive):ti,ab,kw OR (Cognitive Retention Disorder):ti,ab,kw OR (Cognitive Retention Disorders):ti,ab,kw 12204<br><br>#17 (Retention Disorder, Cognitive):ti,ab,kw OR (Memory Loss):ti,ab,kw OR (Memory Losses):ti,ab,kw OR (Age-Related Memory Disorders):ti,ab,kw OR (Age-Related Memory Disorder):ti,ab,kw 2918<br><br>#18 (Age Related Memory Disorders):ti,ab,kw OR (Memory Disorder, Age-Related):ti,ab,kw OR (Memory Disorders, Age-Related):ti,ab,kw OR (Memory Disorders, Age Related):ti,ab,kw OR (Memory Deficits):ti,ab,kw 3687<br><br>#19 (Deficit, Memory):ti,ab,kw OR (Deficits, Memory):ti,ab,kw OR (Memory Deficit):ti,ab,kw OR (Memory Disorder, Semantic):ti,ab,kw OR (Memory Disorders, Semantic):ti,ab,kw 4297<br><br>#20 (Semantic Memory Disorders):ti,ab,kw OR (Semantic Memory Disorder):ti,ab,kw OR (Memory Disorder,	

	Spatial):ti,ab,kw OR (Memory Disorders, Spatial):ti,ab,kw OR (Spatial Memory Disorders):ti,ab,kw 984<br><br>#21 (Spatial Memory Disorder):ti,ab,kw 456<br><br>#22 (Attention):ti,ab,kw OR (Focus of Attention):ti,ab,kw OR (Attention Focus):ti,ab,kw OR (Mental Concentration):ti,ab,kw OR (Concentration, Mental):ti,ab,kw 51182<br><br>#23 (Social Attention):ti,ab,kw OR (Attention, Social):ti,ab,kw OR (Selective Attention):ti,ab,kw OR (Attention, Selective):ti,ab,kw 8618<br><br>#24 (Executive Function):ti,ab,kw OR (Executive Functions):ti,ab,kw OR (Function, Executive):ti,ab,kw OR (Functions, Executive):ti,ab,kw OR (Executive Control):ti,ab,kw 11974<br><br>#25 (Executive Controls):ti,ab,kw 844<br><br>#26 #8 OR #9 OR #10 OR #11 OR #12 OR #13 OR #14 OR #15 OR #16 OR #17 OR #18 OR #19 OR #20 OR #21 OR #22 OR #23 OR #24 OR #25 1164831<br><br>#27 #6 AND #7 AND #26 3196<br><br>#28 (randomized controlled trial):ti,ab,kw AND (RCT):ti,ab,kw 38832<br><br>#29 #27 AND #28 137	

CNKI	(TKA%=艺术疗愈 OR TKA%=艺术治疗 OR TKA%=视觉艺术治疗 OR TKA%=视觉艺术疗法<br>OR TKA%=曼陀罗绘画治疗 OR TKA%=曼陀罗疗法<br>OR TKA%=绘画治疗 OR TKA%=美术治疗<br>OR TKA%=书法治疗 OR TKA%=手工治疗)<br>AND<br>(TKA%=老年 OR TKA%=老年人)<br>AND<br>(TKA%=认知功能 OR TKA%=认知障碍 OR TKA%=轻度认知障碍 OR TKA%=认知能力<br>OR TKA%=记忆力 OR TKA%=记忆障碍<br>OR TKA%=执行力 OR TKA%=执行功能<br>OR TKA%=注意力)	**21**
Wanfang Data	(("艺术疗愈" OR "艺术治疗" OR "视觉艺术治疗" OR "视觉艺术疗法" OR "视觉艺术"OR "曼陀罗绘画治疗" OR "曼陀罗疗法" OR "曼陀罗画"<br>OR "绘画治疗" OR "美术治疗"OR "书法治疗" OR "手工治疗" OR "拼贴" OR "雕塑"))<br>AND<br>("老年" OR "老年人")<br>AND<br>("认知功能" OR "认知障碍" OR "轻度认知障碍" OR "认知能力"<br>OR "记忆力" OR "记忆障碍"<br>OR "执行力" OR "执行功能"<br>OR "注意力")	**19**
CBM	(TM=("艺术疗愈" OR "艺术治疗" OR "视觉艺术治疗" OR "视觉艺术疗法"	**3**

	OR "曼陀罗绘画治疗" OR "曼陀罗疗法"<br>OR "绘画治疗" OR "美术治疗"<br>OR "书法治疗" OR "手工治疗"))<br>AND<br>(TM=("老年" OR "老年人"))<br>AND<br>(TM=("认知功能" OR "认知障碍" OR "轻度认知障碍" OR "认知能力"<br>OR "记忆力" OR "记忆障碍"<br>OR "执行力" OR "执行功能"<br>OR "注意力"))	
VIP Database	(M=("艺术疗愈" OR "艺术治疗" OR "视觉艺术治疗" OR "视觉艺术疗法" OR "视觉艺术"OR "曼陀罗绘画治疗" OR "曼陀罗疗法" OR "曼陀罗画"<br>OR "绘画治疗" OR "美术治疗"OR "书法治疗" OR "手工治疗" OR "拼贴" OR "雕塑"))<br>AND<br>(M=("老年" OR "老年人"))<br>AND<br>(M=("认知功能" OR "认知障碍" OR "轻度认知障碍" OR "认知能力"<br>OR "记忆力" OR "记忆障碍"<br>OR "执行力" OR "执行功能"<br>OR "注意力"))	**11**
