# Supplementary material for: Effects of visual art therapy on cognitive function in older adults: a systematic review and meta-analysis
Source: Front Psychiatry. 2026 Aug 19;17:1828257. doi: 10.3389/fpsyt.2026.1828257 (PMC13534113; doi:10.3389/fpsyt.2026.1828257)
Supplement: Supplementary file 2 [file Table2.pdf]

Supplementary Table S2

Table S2 GRADE Evidence Quality Assessment

Outcome indicator	Participants (studies)	Risk of bias	Inconsistency	Indirectness	Imprecision	Publication bias	SMD/MD (95% CI)	Certainty
Cognitive Function	628 (10 RCTs)	serious[a]	not serious	not serious	not serious	none	0.39 [0.23, 0.54]	⊕ ⊕ ⊕ ○ Moderate[a]
VFT	294 (3RCTs)	serious[a]	not serious	not serious	serious[b]	none	1.21 [0.12, 2.29]	⊕ ⊕ ○○ Low[a,b]
BNT	150 (2RCTs)	serious[a]	not serious	not serious	serious[b]	none	1.74 [0.20, 3.28]	⊕ ⊕ ○○ Low[a,b]
activities of daily living	231 (3RCTs)	very serious[c]	not serious	not serious	serious[b]	none	-0.36 [-0.62, -0.10]	⊕ ○○○ very Low[c,b]

[a] Downgraded one level for risk of bias because blinding of participants and intervention personnel was not implemented, allocation concealment was inadequately reported in some studies, and several studies had a high risk of detection bias.

[b] Downgraded one level for imprecision because of the limited number of studies and participants and the wide confidence intervals, which included effects ranging from trivial to potentially important benefits.

[c]Downgraded two levels for very serious risk of bias because all included studies lacked blinding of participants and intervention personnel, most studies had a high risk of detection bias, and activities of daily living are particularly susceptible to participants' behaviour and assessors' subjective judgement.

Supplementary Table . Summary of findings and certainty of evidence using GRADE
